# The Psychological Pressures of Breast Cancer Patients During the COVID-19 Outbreak in China—A Comparison With Frontline Female Nurses

**DOI:** 10.3389/fpsyt.2020.559701

**Published:** 2020-12-15

**Authors:** Qin Cui, Zhongxiang Cai, Juanjuan Li, Zhongchun Liu, Shengrong Sun, Chuang Chen, Gaohua Wang

**Affiliations:** ^1^Department of Neurology, Renmin Hospital of Wuhan University, Wuhan, China; ^2^Department of Psychiatry, Renmin Hospital of Wuhan University, Wuhan, China; ^3^Nursing Office of Renmin Hospital of Wuhan University, Wuhan, China; ^4^Department of Breast and Thyroid Surgery, Renmin Hospital of Wuhan University, Wuhan, China

**Keywords:** breast cancer, female nurses, COVID-19, anxiety, depression, insomnia, post-traumatic stress disorder

## Abstract

**Objective:** During the outbreak of the COVID-19 epidemic in China, breast cancer (BC) patients and healthcare workers faced several challenges, resulting in great psychological stress. We measured the psychological status of BC patients and female nurses and compared the severity within the two groups at the peak time-point of the COVID-19 outbreak.

**Methods:** A total of 207 BC patients and 684 female nurses were recruited from Wuhan. They completed an anonymous questionnaire online using the most popular social media software in China, WeChat. The psychological status of BC patients and of female nurses was measured using the Chinese versions of the 9-item Patient Health Questionnaire (PHQ-9), the 7-item Generalized Anxiety Disorder scale (GAD-7), the 7-item Insomnia Severity Index (ISI), and the 22-item Impact of Event Scale-Revised (IES-R) for evaluation of post-traumatic stress disorder (PTSD). The differences between the two groups were analyzed.

**Results:** The scores of BC patients and frontline female nurses for the four scales were significantly higher than those of non-frontline female nurses (*P* < 0.001). There were similar scores between BC patients and frontline female nurses for PHQ-9, GAD-7, and IES-R (*P* = 0.789, *P* = 0.101, *P* = 0.158, respectively). Notably, the scores of BC patients for ISI were significantly higher than those of the frontline female nurses (*P* = 0.016). A considerable proportion of BC patients reported symptoms of depression (106/207, 51.2%), anxiety (130/207, 62.8%), insomnia (106/207, 51.2%), and PTSD (73/207, 35.5%), which was more severe than that of female nurses.

**Conclusions:** BC patients experienced great psychological pressure during the COVID-19 outbreak. The incidents of symptomatic anxiety, depression, sleep disorders, and PTSD were significantly comparable to that of frontline female nurses, and episodes of insomnia among BC participants were more serious than for frontline female nurses.

## Introduction

Since the end of 2019, a novel coronavirus, COVID-19, caused by the virus SARS-CoV-2, began to spread in Wuhan, China. This new disease, defined as the Coronavirus Disease 2019 (COVID-19) by the World Health Organization (WHO) on 11 February 2020, spread all over the world (1, 2). The Chinese government placed a lockdown on the epicenter city of Wuhan and quickly conducted powerful and effective measures to fight the pandemic.

All healthcare workers joined in and fought against the pandemic without hesitation. During the initial phase of the pandemic, healthcare workers faced great challenges, such as limited information about COVID-19 and effective drugs, rapidly increasing numbers of patients, and limited resources and protective supplies. At the same time, the non-COVID-19 patients had to discontinue or delay their normal therapy owing to the lockdown policies, limited medical resources, and the predicted increased risk of infection, especially for patients with cancer. Therefore, the outbreak of COVID-19 led to significant increases in the psychological burden of healthcare workers and patients with cancer, especially those with breast cancer (BC).

Our recent investigation showed that healthcare workers suffered great psychological pressure during the COVID-19 pandemic, especially frontline nurses (3). In addition, we evaluated the effects of the pandemic on the psychological status of breast cancer (BC) patients (4). However, there are few comparisons of the psychological status between different groups in the literature. Therefore, in this study, we focused on the severity of psychological problems in BC patients and compared them with that of female nurses in the epicenter of the pandemic, in Wuhan, China. We measured the psychological status of BC patients and nurses at the peak point of the COVID-19 outbreak by using the Generalized Anxiety Disorder Questionnaire (GAD-7), Patient Health Questionnaire (PHQ-9), Insomnia Severity Index (ISI), and Impact of Events Scale-Revised (IES-R) for PTSD evaluation.

## Methods

### Patients

BC patients from the epicenter of COVID-19 in China, Wuhan Hubei Province, were enrolled in this survey study. Female nurses from a tertiary hospital in Wuhan were selected as the control group. The study was sponsored by the Renmin Hospital of Wuhan University. All enrolled patients and nurses signed a digital informed consent form before accessing the questionnaire online. The questionnaire was designed to include demographic characteristics and four validated psychological assessment scales. The clinical features and current treatments were additionally recorded for patients with BC. The four scales included the Generalized Anxiety Disorder Questionnaire (GAD-7), Patient Health Questionnaire (PHQ-9), Insomnia Severity Index (ISI), and Impact of Events Scale-Revised (IES-R). All participants were asked to answer the questionnaire online by using the most popular social media software in China, Wechat. We issued the questionnaire in the WeChat groups from February 1 to 19, 2020, when the daily number of confirmed cases was at its peak. The daily pandemic curve showed that the number of reported cases increased rapidly after January 10, reaching the pandemic peak on February 5, after which point it declined slowly (5).

**Figure 1:**
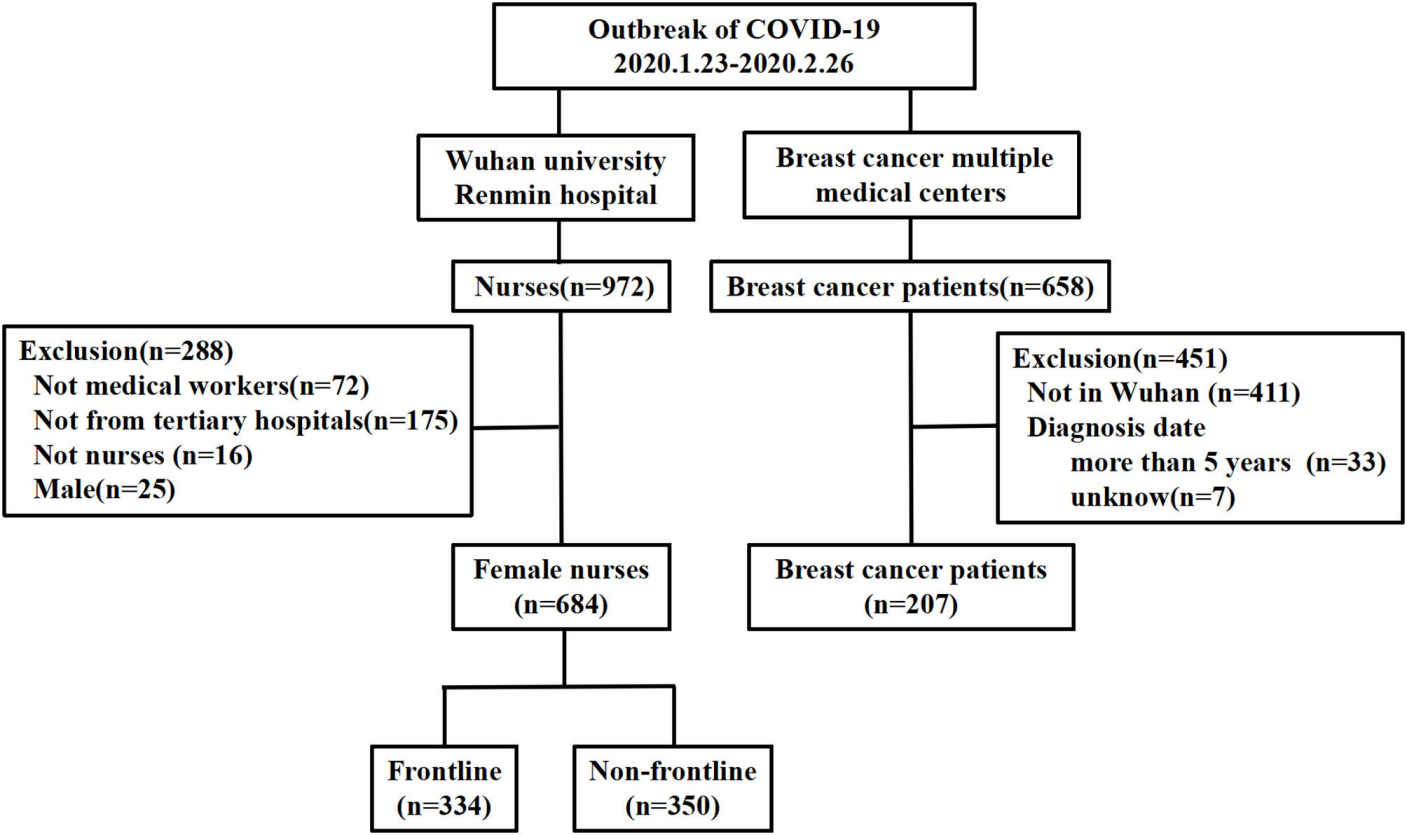
Flowchart of the recruitment of the patients and nurses.

Participants who completed the entire questionnaire in <5 min or more than 60 min were excluded. BC patients whose date of diagnosis was before 2015 and male nurses were excluded. After the evaluation of questionnaires for eligibility, 891 participants were enrolled for analysis, including 207 BC patients and 684 female nurses. The female nurses were divided into a frontline and a non-frontline group. The nurses from the emergency department, fever clinics, or the medical unit for COVID-19 patients were identified as frontline nurses, and the others were non-frontline nurses. The flowchart of patient and nurse selection is shown in Figure 1. This study protocol was approved by the Institutional Ethics Committee of Renmin Hospital of Wuhan University.

### Psychological Status Evaluation

We used four questionnaire scales to evaluate the psychological status of BC patients and nurses during the epidemic, including the PHQ-9, GAD-7, ISI, and IES-R. The validity and reliability of depression on the PHQ-9 scale (6, 7) and generalized anxiety on the GAD-7 scale (8, 9) have been demonstrated previously. The PHQ-9 with nine items and the GAD-7 with seven items were rated from 0 (“almost never”) to 3 (“almost always”). Based on the scores obtained on these scales, the severity of anxiety or depression for participants was divided into normal (0–4), mild (5–9), moderate (10–14), and severe (>15). The respondents whose scores were higher than 4 in PHQ-9 or GAD-7 were thought to have depressive or anxiety symptoms.

The ISI (10) is a seven-item instrument for insomnia assessment utilizing a 5-point Likert scale (0–4, not at all to extremely). The insomnia status was divided into no sleep difficulties (0–7), mild (8–14), moderate (15–21), and severe insomnia (22–28). The respondents whose scores were higher than 7 in ISI were thought to have sleep problems.

The IES-R scale (11) was used to assess Post-traumatic Stress Disorder (PTSD) symptoms based on DSM-IV criteria. Each item was rated using a five-point Likert scale ranging from 0 (not at all) to 4 (very much), for a total score ranging from 0 to 88. Participants with a score of more than 34 were defined as having PTSD.

### Statistical Analysis

All statistical analyses were carried out using IBM SPSS Statistics (Version 26.0). One-way ANOVA, independent-samples *T*-test, and Chi-square-test were used to compare differences in the psychological status of BC patients and female nurses, based on the PHQ-9, GAD-7, ISI, and IES-R. A corresponding 95% confidence interval (CI) was calculated, and the statistical significance level was set at *P* < 0.05.

## Results

### Characteristics of BC Patients and Female Nurses in Wuhan

A total of 207 BC patients were collected in this study, including 113 cases (54.6%) within 1 year of their BC diagnosis. The majority of the patients were married (81.2%), younger than 55 years old (72.0%), had no bachelor's or higher degree (66.2%), and earned an annual income of < $15,000 (154/207; 74.9%). Most of the patients identified themselves as having presented with a good or average physical condition in the past. There were 73.4% of participants with early-stage BC disease, 59.9% who reported a history of prior breast surgery (194/207; 93.7%), and 79.7% who were advised to undergo BC treatment during COVID-19. The baseline characteristics of the BC patients are shown in Table 1.

**Table 1:** The baseline characteristics of breast cancer patients and nurses in Wuhan.

		**No. (207)**	**%**
Age (years)	<40	43	20.8
	40–55	106	51.2
	>55	58	28.0
Highest level of education	Elementary school or less	10	4.8
	Middle school	36	17.4
	High school	91	44.0
	Bachelor's degree or higher	70	33.8
Marital status	Unmarried	15	7.2
	Married	168	81.2
	Divorced/widowed	24	11.6
Annual income (US dollars)	< $7500	91	44.0
	$7500–$15,000	64	30.9
	$15,000–$43,000	46	22.2
	>$43,000	6	2.9
General health condition by self-identification	Well	74	35.7
	Average	78	37.7
	Poor	55	26.6
Someone infected with COVID-19 around breast cancer patients	Yes	24	11.6
	No	18	8.7
	N/A	165	79.7
Breast cancer diagnosis time	Within 1 year	113	54.6
	More than 1 year	94	45.4
Breast cancer stage	Early	124	59.9
	Advanced	40	19.3
	Unknown	43	20.8
Molecular subtype of breast cancer	TNBC	36	17.4
	Luminal	55	26.7
	HER2	68	32.8
	Unknown	48	23.1
History of breast cancer surgery	Yes	194	93.7
	No	13	6.3
Recommend anti-cancer therapy	Yes	165	79.7
	No	42	20.3
Discontinued anticancer therapy	Endocrine therapy	103	53.4
	Targeted therapy	30	15.5
	Chemotherapy	47	24.4
	Radiotherapy	5	2.6
	Traditional Chinese medicine	8	4.1

*N/A, Not provided or not available*.

A total of 684 questionnaires completed by female nurses were received. Three hundred and thirty-four (48.4%) were from the frontline. Most of the nurses had a college education (96.9%) and identified themselves as being in good or average health (96.8%) in the past. The majority of nurses (86.4%) were <40 years old. More than half of the nurses were married (53.2%). Nurses with a primary professional title constituted 57.9% and 58.3% felt uncertain about fighting against the pandemic. The baseline characteristics of the nurses are shown in Table 2.

**Table 2:** The characteristics of female nurses in Wuhan during the outbreak.

		**Female nurses** ***N*** **=** **684**		**Frontline female nurses** ***N*** **=** **334**		**Non-frontline nurses** ***N*** **=** **350**	
		**No**.	**%**	**No**.	**%**	**No**.	**%**
Age/years	18–25	171	25.0	87	26.0	84	24.0
	26–30	236	34.5	115	34.4	121	34.6
	30–40	184	26.9	102	30.5	82	23.4
	>40	93	13.6	30	9.0	63	18.0
Marital status	Unmarried	310	45.3	163	48.8	147	42.0
	Married	364	53.2	166	49.7	198	56.6
	Divorced or widowed	10	1.5	5	1.5	5	1.4
Highest level of education	Junior college degree	4	0.6	2	0.6	2	0.6
	Bachelor's degree	648	94.7	321	96.1	330	94.2
	Master's degree or higher	32	4.7	11	3.3	18	5.2
Professional title	None	94	13.7	46	13.8	48	13.7
	Primary	396	57.9	204	61.6	192	54.9
	Junior	185	27.0	79	23.7	106	30.3
	Senior	9	1.3	5	1.5	4	1.1
Change of physical condition by self-identification	Similar	477	69.7	214	64.1	263	75.1
	Worse	207	30.3	120	35.9	87	24.9
Uncertainty of fighting against the epidemic	Yes	399	58.3	211	63.2	188	53.7
	No	285	41.7	123	36.8	162	46.3

### The Scores of BC Patients and Female Nurses in the Four Scales

**Figure 2:**
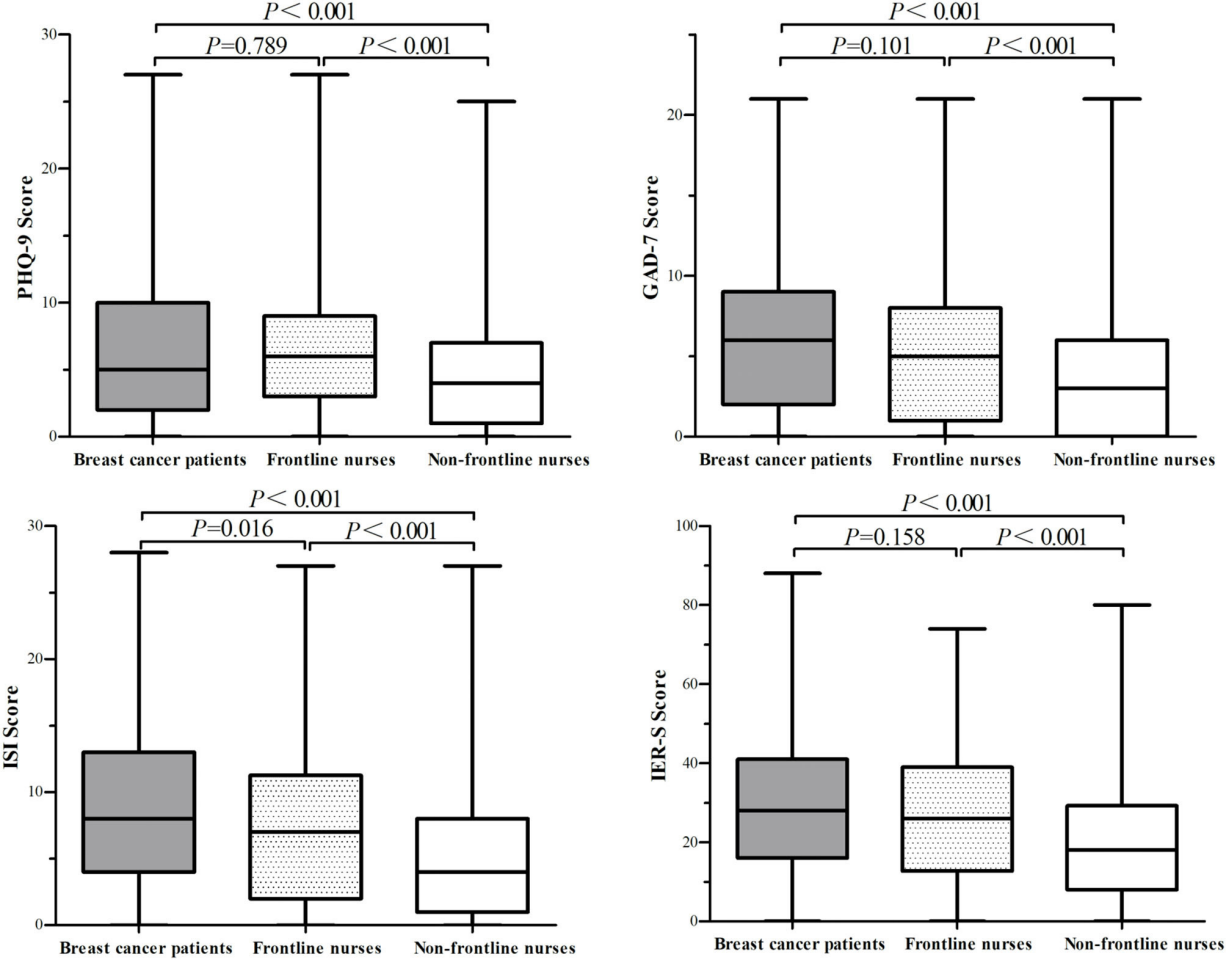
The scores of the breast cancer patients, frontline nurses, and non-frontline nurses in PHQ-9, GAD-7, ISI, and IES-R. PHQ-9, Patient Health Questionnaire; GAD-7, Generalized Anxiety Disorder Questionnaire; ISI, Insomnia Severity Index; IES-R, Impact of Events Scale-Revised.

The scores of the BC patients in PHQ-9, GAD-7, ISI, and IES-R were 6.56 ± 6.044, 6.30 ± 4.879, 8.99 ± 6.359, and 29.12 ± 17.656 respectively; the scores of the frontline female nurses in PHQ-9, GAD-7, ISI, and IES-R were 6.68 ± 5.378, 6.53 ± 4.946, 7.77 ± 6.221, and 27.05 ± 17.377 respectively; the scores of the non-frontline female nurses in PHQ-9, GAD-7, ISI, and IES-R were 4.53 ± 4.305, 3.92 ± 4.127, 5.33 ± 4.378, and 20.08 ± 15.021 respectively. The psychological scores of the BC patients, frontline female nurses, and non-frontline female nurses for the four questionnaires are shown in Figure 2. Scores from the four scales administered to the BC patients were all significantly higher than for the scores of female nurses on the PHQ-9, GAD-7, ISI, and IES-R (*P* = 0.035, *P* < 0.0001, *P* < 0.0001, and *P* < 0.0001, respectively). The scores of BC patients and those of the frontline female nurses for the four scales were significantly higher than those of non-frontline female nurses (*P* < 0.001). There were similar scores between BC patients and frontline female nurses for PHQ-9, GAD-7, and IES-R (*P* = 0.789, *P* = 0.101, *P* = 0.158, respectively). Notably, the scores of BC patients on the ISI were significantly higher than those of frontline nurses (*P* = 0.016).

### The Proportion of Psychological Problems Identified on the Four Scales for BC Patients and Female Nurses

The proportions of psychological problems identified on the four scales in BC patients and female nurses are shown in Table 3. More than half of BC patients and frontline female nurses revealed incidents of depression and anxiety. The scores associated with these factors were significantly higher than those of the non-frontline female nurses. The sleep problems for BC patients and frontline female nurses were significantly higher than those of the non-frontline female nurses. More than one-third of BC patients and frontline female nurses endured a significantly higher proportion of PTSD symptoms than did the non-frontline female nurses.

**Table 3:** The abnormal proportion of four scales in nurses and breast cancer patients.

		**Frontline nurses** ***N*** **=** **334**		**Non-frontline nurses** ***N*** **=** **350**		**Breast cancer patients** ***N*** **=** **207**		
		**No**.	**%**	**No**.	**%**	**No**.	**%**	***P-*value**
PHQ-9	Normal	132	39.5	190	54.3	101	48.8	0.001
	Abnormal	202	60.5	160	45.7	106	51.2	
GAD-7	Normal	148	44.3	213	60.9	77	37.2	<0.001
	Abnormal	186	55.7	137	39.1	130	62.8	
ISI	Normal	180	53.9	245	70.0	101	48.8	<0.001
	Abnormal	154	46.1	105	30.0	106	51.2	
IER-S	Normal	223	66.8	284	81.1	134	64.7	<0.001
	Abnormal	111	33.2	66	18.9	73	35.3	

*P-value was calculated using the Chi-square-test*.

*PHQ-9, Patient Health Questionnaire; GAD-7, Generalized Anxiety Disorder Questionnaire; ISI, Insomnia Severity Index; IES-R, Impact of Events Scale-Revised*.

## Discussion

During the COVID-19 outbreak, rapidly rising numbers of infected cases put both the local healthcare system and the citizens in Wuhan, as the epicenter of COVID-19 in China, under tremendous stress. The Chinese government quickly took powerful and effective measures to fight against the COVID-19 pandemic, such as the lockdown of Wuhan city, integration of personal and medical resources, and the construction of Fangcang shelter hospitals (12). Under this unique circumstance, patients with cancer and healthcare workers experienced significant mental stress. In this study, we focused on the psychological status of BC patients and female nurses in Wuhan city at the peak time-point of the COVID-19 outbreak. Our survey showed that more than half of BC patients had symptoms of depression, anxiety, and insomnia, and over one-third of BC patients endured distress. The proportion of psychological problems in BC patients was comparable to that of frontline female nurses.

Because of limited medical resources, a higher risk of infection with COVID-19, and the possibility of experiencing worse outcomes after infection, the BC patients had to delay or discontinue their planned anti-cancer treatments, increasing the psychological pressure on these individuals. Our study showed that more than half of BC patients suffered from depression (51.2%), anxiety (62.8%), and sleep problems (51.2%), and over one-third of BC patients experienced PTSD symptoms (35.3%). These proportions of psychological problems were higher than those from previous reports in normal situations. BC patients already have a lot of psychological stress as a result of the diagnosis and treatment of the tumor in their bodies. Recent systematic studies have summarized the prevalence of psychological problems in BC patients (13, 14). The results showed that nearly one-third (32.2%) of BC patients experienced depression (13) and nearly 10% of patients (9.6%) had PTSD (14). However, the prevalence of depression, anxiety, insomnia, and PTSD displayed great discrepancies from previous studies (13–17), partly due to the utilization of different definitions, measurements, populations, and the timing of assessments. Therefore, we compared the severity of the psychological problems of BC patients with those of female nurses in the same place and during the same period of the pandemic.

In the face of COVID-19, healthcare workers have taken an active part in fighting the pandemic, regardless of their own safety. Many health care workers suffered great psychological pressure during the outbreak of the pandemic. Many studies have shown that healthcare workers, especially nurses, endured significantly high psychological problems during the outbreak of SARS or MERS (18–21). In our recent study, we assessed the magnitude of mental health outcomes and associated factors among 1,257 healthcare workers for COVID-19 patients in multiple regions of China (3). The results showed that a considerable proportion of healthcare workers reported experiencing symptoms of depression, anxiety, insomnia, and distress. This was especially true for frontline female nurses from the epicenter, Wuhan. We accordingly deduced that frontline female nurses in Wuhan are under the most severe psychological pressure. In this survey, more than half of frontline female nurses suffered from depression (202/334, 60.5%) and anxiety (186/334, 55.7%), and over one-third of female nurses experienced sleep problems (154/334, 46.1%) and PTSD (111/334, 33.2%). The proportions of symptomatic depression, anxiety, insomnia, and PTSD in frontline female nurses were significantly higher than those in non-frontline female nurses. However, our results showed that BC cancer patients were under psychological pressure comparable to that of the frontline female nurses at the peak of the COVID-19 outbreak in Wuhan, China. This is the first report offering a direct comparison of psychological status between patients and nurses during the pandemic. BC patients, in fact, were found to suffer worse symptoms of insomnia than female frontline nurses. These results indicate that more attention should be paid to the psychological problems experienced by BC patients and that more effective intervention measures need to be taken during future epidemics.

There were significant differences in age, marital status, and levels of education between the BC patients and female nurses. They were all women living in Wuhan, who had to go to hospitals while being at a greater risk of infection with COVID-19. Nurses are at the forefront of fighting the pandemic, and their psychological state easily attracts public attention. By comparing the psychological status of BC patients with that of nurses, we were able to obtain a greater understanding of the psychological state of the BC patients. It is important to pay attention to the psychological status of people with chronic illnesses during the outbreak of a pandemic.

During the outbreak, a number of modifications to standard treatment paradigms were implemented for BC patients. However, in addition to obtaining support through online or offline services from professional health care workers (22), necessities from social volunteers, BC patients also need to receive more support from their families. This is crucial because most remained at home during the pandemic. Several reports have demonstrated the importance of family support for patients. A prospective study with a long-term follow-up for patients showed that family support was associated with both low levels of, and quick improvement from, depression (23). For BC patients aged more than 55 years, family support from adult children might decrease their levels of anxiety and depression (24). Kamen et al. (25) showed that family support was related to less severe insomnia at baseline in BC patients. Additionally, one previous study demonstrated that family support could avoid or alleviate certain mood difficulties in BC patients in the Chinese population (26). Therefore, people need to be educated regarding the importance of providing support for BC family members, especially during epidemic outbreaks.

We acknowledge some shortcomings in our study. First, our study is a cross-sectional study that only extracts data from one point in time. The changes in the psychological status of BC patients should be investigated at different periods of the pandemic. Second, the sample size of BC patients was small. Third, the direct comparison of psychological pressures between BC patients and nurses might not be appropriate because of different influencing factors in the two distinct groups. However, our findings indicated that when BC patients were forced to delay or discontinue treatment due to the pandemic, their psychological pressures increased greatly. The differences in psychological status among other populations, such as the normal population, BC patients more recently diagnosed from other areas, and patients with other chronic diseases, should be evaluated in future. Moreover, effective measures for alleviating the psychological pressures of BC patients, both during and after the pandemic, should be investigated.

## Conclusions

In summary, our study showed that BC patients were under great psychological pressure as compared to frontline female nurses in the COVID-19 epicenter, Wuhan, China. BC patients suffered worse incidents of insomnia than frontline female nurses who treated COVID-19 patients. These results indicate that effective measures should be taken to alleviate the psychological problems of BC patients during pandemics. The importance of family support in relieving psychological stress in these patients is also emphasized.

## Data Availability Statement

The raw data supporting the conclusions of this article will be made available by the authors, without undue reservation.

## Ethics Statement

The studies involving human participants were reviewed and approved by the Institutional Ethics Committee of Renmin Hospital of Wuhan University. The ethics committee waived the requirement of written informed consent for participation.

## Author Contributions

QC: validation, formal analysis, writing—original draft, and visualization. ZC: conceptualization, methodology, validation, formal analysis, resources, data curation, and supervision. ZL: methodology, project administration, and funding acquisition. JL: formal analysis and investigation. SS: investigation, resources, and project administration. CC and GW: conceptualization, writing-review and editing, visualization, supervision, and funding acquisition. All authors contributed to the article and approved the submitted version.

## Conflict of Interest

The authors declare that the research was conducted in the absence of any commercial or financial relationships that could be construed as a potential conflict of interest.
